# Endoscopic Submucosal Dissection of the Angiolipoma at Hypopharynx-Esophageal Introitus

**DOI:** 10.1155/2020/3581267

**Published:** 2020-02-14

**Authors:** Lu Liu, Feng Miao, Hai-Mei Guo, Nuo Li, Shu-Hua Jiao, Shuang Cai, Peng-Liang Liu, Shan-Shan Zhang, Jia Ma, Yang Weng, Ying Sun, Yin-Si Tang, Feng Zhao, Yan Zheng, Shen Zhang, Yan Yang, Yang Yu, Lei Tian, Zhi-Feng Zhao

**Affiliations:** ^1^Department of Gastroenterology, The Fourth Affiliated Hospital of China Medical University, Shenyang, 110000 Liaoning Province, China; ^2^Department of Gastroenterology, The First Affiliated Hospital of Jinzhou Medical University, Jinzhou, 121001 Liaoning Province, China

## Abstract

Angiolipoma in the region of the hypopharynx-esophageal introitus is a rare occurrence. Surgical treatment was performed in the few cases reported in the literature. Endoscopic submucosal dissection (ESD) is a minimally invasive treatment for hypopharyngeal and esophageal lesions. Our objective was to evaluate the feasibility, safety, and efficacy of ESD for treatment of angiolipoma at the hypopharynx-esophageal introitus. The patients with submucosal tumors at the hypopharynx-esophageal introitus were diagnosed as angiolipoma by preoperative evaluation with endoscopy, endoscopic ultrasonography, and computed tomography (CT). The patients who were diagnosed with angiolipoma agreed to undergo endoscopic submucosal dissection. Under general anesthesia and endotracheal intubation, ESD was used to remove the lesions. Preoperative, intraoperative, and postoperative data were collected and analyzed to evaluate the feasibility, safety, and effectiveness of endoscopic submucosal dissection. From January 2013 to December 2018, 6 cases of angiolipoma were treated with ESD with a success rate of 100%. The average operation time was 107.0 ± 69.4 minutes. Intraoperative blood loss is the main risk. Endoscopic thermocoagulation successfully stopped bleeding in all cases. Pharyngeal pain and painful swallowing were the main clinical signs. There was no stricture at the hypopharynx-esophageal introitus after the operation. ESD treatment of angiolipoma at hypopharynx-esophageal introitus is feasible, safe, and effective.

## 1. Introduction

Angiolipoma is one of the most common benign soft tissue tumors. In a few cases, familial heredity was observed [1]. It commonly occurs in young men's arms and trunk, and it can also occur in the oral cavity and digestive tract. Submucosal solitary or intraluminal polypoid tumors characterize angiolipomas of the gastrointestinal tract. Surgical excision is the primary treatment. Six cases of angiolipoma in the region of the hypopharynx-esophageal introitus were treated with endoscopic mucosal dissection (ESD) in our hospital.

## 2. Materials and Methods

### 2.1. Patients

In total, 6 patients with angiolipoma located at the hypopharynx-esophageal introitus in patients were treated in the Department of Gastroenterology, the Fourth Affiliated Hospital of China Medical University between July 2013 and January 2018. The average age of the patients was 52.7 years (27-85 years). There were four males and two females. The main clinical manifestations were dysphagia in 4 cases and intermittent chest pain in 2 cases. Six patients failed to complete gastroscopy in the other hospitals. In one case, the gastroscopy failed to pass through the hypopharynx, while in 3 cases, the gastroscopy entered the esophageal introitus but could not progress. In the remaining 2 cases, the gastroscope was progressed to the middle third of the esophagus before the evaluation was terminated. Routine gastroscopy was successfully performed in these 6 patients in our outpatient department with the esophageal tumor duly characterized in each case. The tumor was considered to have originated from the hypopharynx. The mean tumor size was 5.63 cm (3.8-30 cm).

The patients underwent preoperative gastroscopy, ultrasonic gastroscopy, and CT examination with a presumptive diagnosis of lipoma at hypopharynx-esophageal introitus made based on clinical experience. After analysis in each separate case, endoscopic mucosal dissection was selected for these patients. All patients were informed of the possible complications of the operation. Additionally, they were informed about the possibility of additional procedures to manage complications or postoperative pathologic findings.

### 2.2. Preoperative Instruments

Preoperative instruments included Olympus CV 260sl, Olympus GIF Q260J, and Distal Attachment Cap (Shang Xian Co., Ltd.).

All ESD procedures were performed by the same surgeon (Zhao ZF). Patients underwent endotracheal intubation under intravenous general anesthesia.

### 2.3. Therapeutic Methods

ESD: after submucosal injection of normal saline containing methylene blue and hyaluronic acid at the pedicle of the tumor, the mucosa and submucosa layers were gradually dissected with incision knife and the tumor was completely dissected. Intraoperative blood vessel bleeding was stopped by means of thermocoagulation forceps, and hemostasis of the dissected wound surface was achieved by thermocoagulation. All wounds were left open without suturing ([Supplementary-material supplementary-material-1], [Supplementary-material supplementary-material-1], [Supplementary-material supplementary-material-1], [Supplementary-material supplementary-material-1], [Supplementary-material supplementary-material-1]).

## 3. Results and Discussion

### 3.1. Gastroscope

Gastroscopy showed that the left side of hypopharynx was difficult to progress in all cases, so we passed the endoscope through the right side of hypopharynx to enter the esophagus. Following successful gastroscopy in all patients, the mean tumor size was determined to be 5.63 cm (3.8-30 cm).

**Figure 1:**
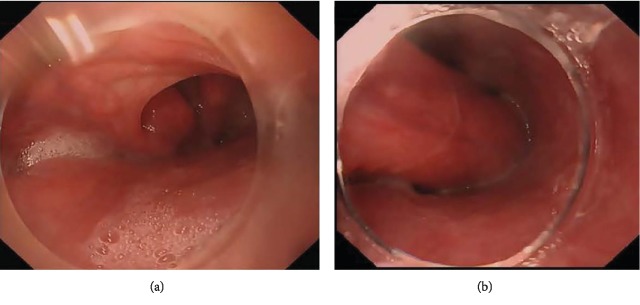
A large protruded tumor (30 cm) was seen in the esophageal lumen; its pedicle originated from the hypopharynx and prolapsed into the esophagus.

**Figure 2:**
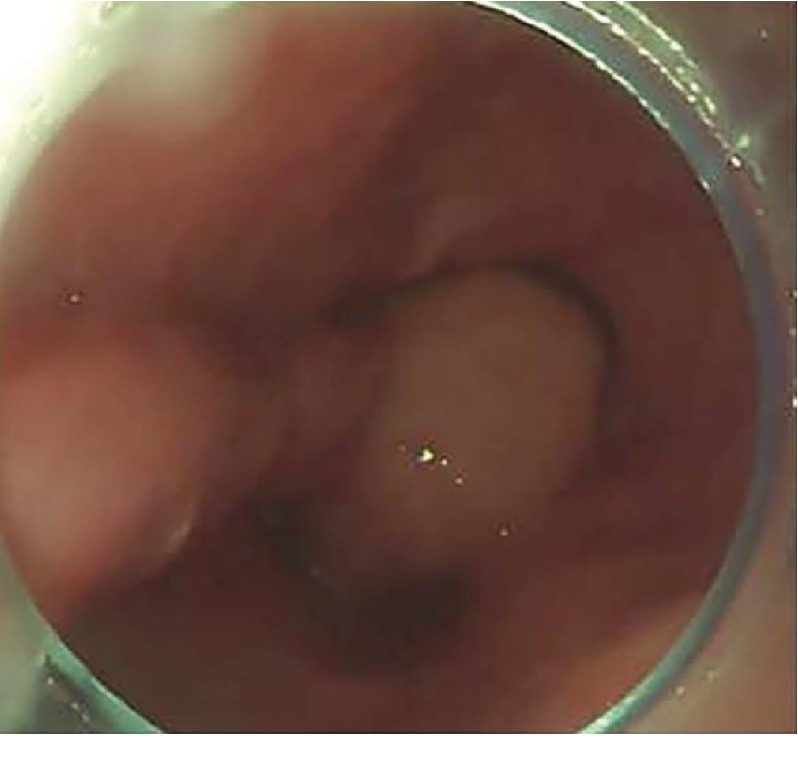
The tumor prolapsed into the esophagus, and the esophageal lumen was dilated. The surface of the tumor was smooth and yellowish-white in color.

**Figure 3:**
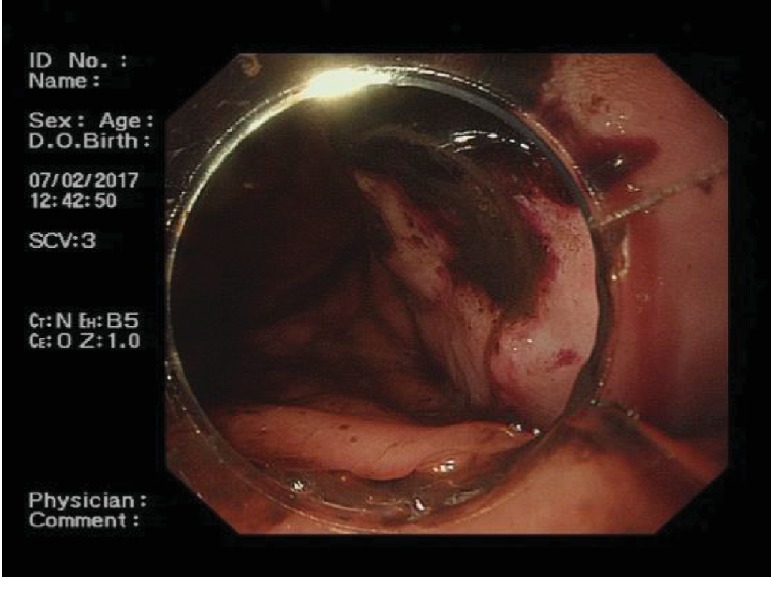
The distal end of the tumor was in the stomach where a large amount of old accumulated blood was found. Bleeding ulcerations were observed on the surface of the tumor.

In total, 6 patients with angiolipoma originated from the hypopharynx-esophageal introitus region were treated in our hospital, including 4 cases with the tumor pedicle at the hypopharynx (Figure 1) and 2 cases with the tumor pedicle in the esophagus using gastroscopy with distal attachment cap; all 6 cases were with tumor pedicle. There was 1 case of a large tumor (30 cm), which prolapsed into the stomach, 1 case into the lower third of the esophagus, 2 cases into the middle-third of the esophagus, and 2 cases into the esophageal introitus. Gastroscopic findings of the tumors: the tumors caused the hypopharynx to shift to the right side, the esophageal introitus was pulled by the tumor pedicle, and the surface of the tumors showed a large number of dilated veins. The esophageal lumen was dilated, and the esophageal wall was normal. The surface of the tumor was smooth and yellowish-white in color (Figure 2). The surface was tough and was slightly deformed by squeezing. In the case that the tumor prolapsed into the stomach, the tumor surface was accompanied by ulceration and bleeding, and coffee-like old bleeding was seen in the stomach cavity (Figure 3). In all the cases, no biopsy was taken under gastroscope (Table 1).

### 3.2. Endoscopic Ultrasonography

**Figure 4:**
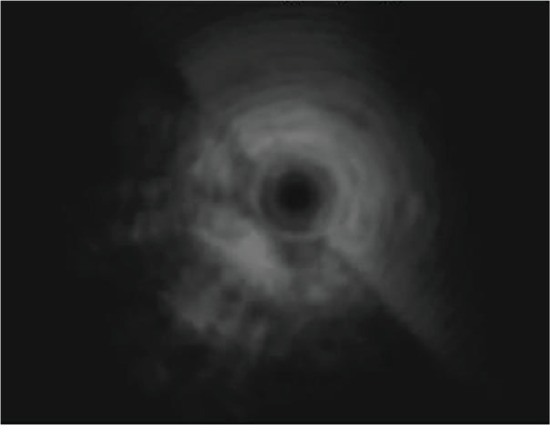
EUS showed both a good mucosal layer, an irregular submucosal, muscle-like area, and slightly hyperechoic areas.

We initially performed radial scanning and linear endoscopic ultrasonography, all of which failed. Among them, 4 cases had severe pain when entering the esophageal introitus, so the examination was canceled. In the remaining 2 cases, entry through the esophageal introitus was challenging. All patients successfully completed routine gastroscopy combined with microprobe endoscopic ultrasonography (EUS). EUS (Figure 4) revealed a good mucosal layer, irregular submucosal, muscle-like area, and several slightly hyperechoic areas. However, microprobe EUS images were severely attenuated and the image quality was low.

### 3.3. CT Examination

**Figure 5:**
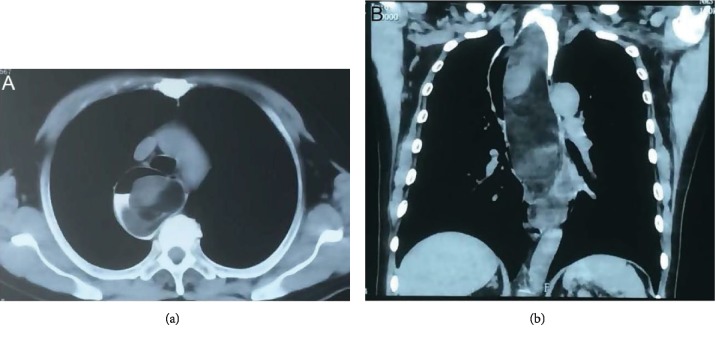
The esophagus was dilated; the right side of the esophageal lumen showed a large soft tissue mass with an irregular shape, a smooth surface with nonuniform density.

All the 6 cases underwent imaging by noncontrast CT (Figure 5). CT revealed that the tumor originated from the hypopharynx-esophageal introitus in 1 case, while the origin of the tumors in the other 5 cases was not clearly observed. The right side of the esophageal cavity showed a large soft tissue mass with an irregular shape, smooth surface, nonuniform, mostly fat-like low density, and mixed with other components. In the patient with a large tumor, the tumor extended from the hypopharynx-esophageal introitus to the stomach, and high vascularity was observed in the tumor.

### 3.4. Endoscopic Submucosal Dissection

Combining the imaging and gastroscopy results, we concluded on the diagnosis of angiolipoma at the hypopharynx-esophageal introitus, and after much deliberation, chose to perform endoscopic submucosal dissection. Hemorrhage occurred in all 6 cases, mainly during injection and incision. During the operation, 2 cases suffered large blood vessel hemorrhage due to increased vascularity on the tumor surface. Thermocoagulation forceps-assisted hemostasis was successful, and the intraoperative blood loss was about 100 ml in these two cases. Two cases (case 1 and case 2) with large tumors were treated with a snare to cut the tumor into blocks. About one-fifth of the tumor was removed for examination. The repeated attempts to take out the resected tumor failed, and the attempt to push the whole tumor into the stomach also failed. Therefore, we chose to use incision knife and snare to cut the tumor into blocks and pushing the parts into the stomach, so that the tumor was exposed under the action of gastric acid and then corroded. The tumors were removed after segmental incision in 2 cases (cases 4 and 5). The whole tumors were completely removed in 2 cases (cases 3 and 6). Surgical duration was 50-240 minutes (107.0 ± 69.4 minutes). The wound was left open about 1-1.5 cm. All the tumors were resected successfully. During the reexamination using gastroscopy, the exposed tumors were excreted from the body; no residues were found in the stomach (Table 2).

### 3.5. Postoperative Pathology

**Figure 6:**
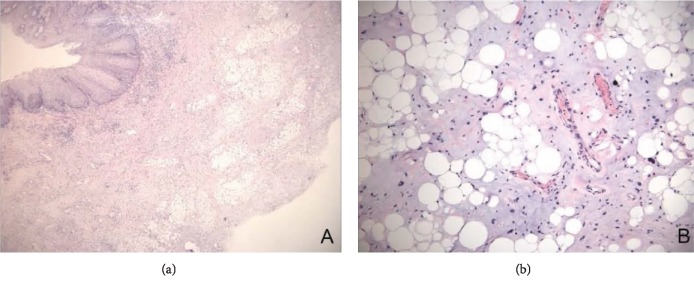
In addition to mature adipocytes, there are hyperplastic capillaries and endothelial cells in the tissue. Interstitial collagen fibers were homogeneous, pale, and eosinophilic, with no obvious inflammatory reaction.

Immunohistochemical staining was performed. As shown in Figure 6, the tumor tissue has a distinct capsule, the cut surface was yellow, and its edge was reddish due to its vascular component. In addition to mature adipocytes, there are hyperplastic capillaries and endothelial cells in the tissue. Interstitial collagen fibers were homogeneous, pale eosinophilic, with no obvious inflammatory reaction. The pathology diagnosis for all cases was fibroangiolipoma.

### 3.6. Follow-Up

**Figure 7:**
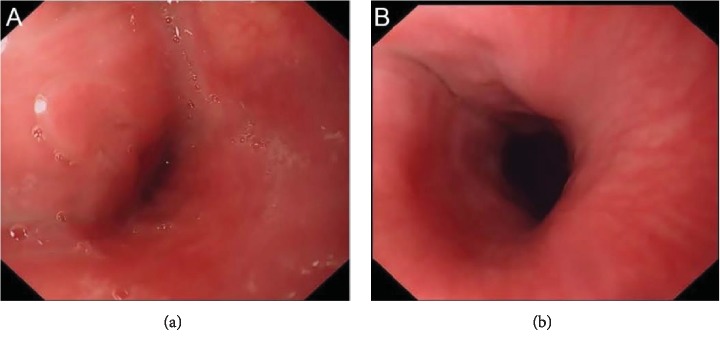
One year after operation, scar formation at esophageal introitus was found by gastroscopy. No stricture was found in the esophageal lumen of each patient.

The patients were observed for 1-week after ESD in hospital, then followed up at two months and one year after ESD. During the 1-week observation, we used the 0-10 Numeric Pain Rating Scale (NPRS) to measure the pain intensity—0: no pain, 1-3: mild pain, 4-6: moderate pain, and 7-10: severe pain. Two cases showed postoperative pharyngeal pain; a further two cases showed pain when drinking water; all pain levels were tolerable (pain scores < 3). One year after the operation, scar formation at the esophageal introitus was found by gastroscopy in six patients. (Figure 7). No stricture was found in the esophageal lumen of each patient, and the patients' food intake was not affected. Conversely, there was no resistance when passing the endoscope during repeat gastroscopy. The esophageal lumen returned to normal in 4 cases, with esophageal dilatation notably improved in one case, while showing no obvious improvement in the remaining case. The esophageal mucosa was normal in all cases.

## 4. Discussion

### 4.1. Angiolipoma

Angiolipoma is a distinct morphological variation of a lipoma, accounting for 5-7% of lipomas [2, 3]. Its histological features include mature adipose tissue and scattered proliferating vessels [4]. Thrombosis is rare in patients with gastrointestinal angiolipoma [5]. Familial heredity was observed in a few cases of angiolipoma. Familial angiolipomatosis has a predominantly autosomal-recessive inheritance, but some studies also show an autosomal dominant mode of inheritance [1]. Gastrointestinal angiolipoma can occur in the oral cavity [6, 7], hypopharynx [8, 9], esophagus [10], stomach [5], duodenum [3], ileum [11], and colorectum [12, 13]; a few cases have been reported in these positions, respectively. Gastrointestinal angiolipomas are characterized by indigestion, intestinal obstruction, intussusception, and bleeding [3, 11, 12].

Angiolipoma occurring at the hypopharynx-esophageal introitus is extremely rare in clinical practice. A literature search of “hypopharynx-esophagus lipoma” and “hypopharyngeal lipoma” revealed 1 case of pharyngeal lipoma [8], 1 case of hypopharyngeal lipoma [9], and 1 case of esophageal lipoma [10]. Six patients with angiolipoma at the hypopharynx-esophageal introitus were treated in our hospital during the past 5 years. The results showed that the main clinical manifestations of angiolipoma at the hypopharynx-esophageal introitus were dysphagia and intermittent chest pain. The large tumors often prolapse into the esophagus, with the hypopharynx deformed by traction, and the esophageal cavity frequently dilated due to the space-occupying tumor. As for larger tumors, the distal end may prolapse into the gastric cavity. The distal end of the tumor is often with surface rupture, ulcer, and bleeding, and if it reached the stomach, the patient would manifest symptoms indicative of gastrointestinal bleeding and anemia. Angiolipoma at the hypopharynx-esophageal introitus needs to be differentiated from hypopharyngeal carcinoma, esophageal cancer, and stromal tumor.

### 4.2. Diagnostic Methods

#### 4.2.1. Gastroscopy

Angiolipomas at the hypopharynx-esophageal introitus often deform the hypopharynx by traction and occupy the esophageal lumen. Gastroscopy is generally difficult. All six patients failed to complete gastroscopy in the other hospitals, the gastroscopy failed to pass through the hypopharynx in 1 case, the gastroscopy entered esophageal introitus but could not progress in 3 cases, and the gastroscopy arrived the middle third of the esophagus and terminated the examination in 2 cases. The routine gastroscopic examination in our hospital is also difficult, but it was successful in all 6 patients. All gastroscopes succeeded in entering the esophagus along the right side of the hypopharynx. The left side of hypopharynx is difficult to enter, and the right side of the hypopharynx is easy to enter, which is closely related to the distortion of the hypopharynx-esophageal introitus caused by the traction of the prolapsed tumor. Because the tumor completely occupies the esophageal cavity, it is difficult to progress in the esophagus, which is mainly manifested in a small gap, high resistance, and distortion of the esophagus. Distal attachment cap-assisted examination is helpful for the observation of the origin, pedicle range, and endoscopic observing of the tumor. The endoscopic manifestations of the tumors mainly include the following aspects: (1) The hypopharynx is pulled and shifted, and the hypopharynx mucosa was pulling the mucosal bridge, and with the traction directed towards the right side in all cases. (2) The traction change of esophageal introitus by the pedicle of the tumor was seen, the pedicles were located at the left and anterior walls of the esophagus, and the surface of the tumors showed a large number of dilated veins. (3) The esophageal lumen is filled with a tumor; the esophagus is enlarged; the gastroscopy can squeeze out the gap between the tumor and the esophageal wall. (4) The mucosa on the tumor surface is smooth and slightly yellowish-white, and the esophageal wall has no abnormal changes. (5) The tumors were with a firm texture and can be squeezed to slightly deform. (6) In the case that the tumor prolapsed into the stomach, the tumor surface was accompanied by rupture, ulceration, and bleeding, and coffee-like old bleeding was seen in the stomach cavity. The presurgical diagnosis and differential diagnosis of angiolipoma mainly rely on endoscopic manifestations, ultrasonic endoscopic changes, CT, and postoperative pathology. Because angiolipoma is rich in blood supplies and it is difficult to get the tissue from the biopsy at the mucosal surface, hence endoscopic biopsy is not recommended [9], so no biopsy was performed on these patients.

#### 4.2.2. EUS

EUS can preoperatively judge the nature of the tumor by evaluating the components of the tumor, which is of great significance to diagnosis and treatment. The results of this case series demonstrated that radial scanning and linear endoscopic ultrasonography were difficult to perform. Among the 6 cases of unsuccessful radial scanning endoscopic ultrasonography, 4 cases were terminated due to severe pain, and it was difficult to enter the esophageal introitus in 2 cases. The main reason for the failure of endoscopy progression is that the deformation of hypopharynx due to traction and tumor occupying the esophageal lumen, which results in the difficulty for radial scanning and linear endoscopy entering the esophagus. The main cause of pain in patients during endoscopy progression is that the tumor pulls the tissue of esophageal introitus, and the endoscopy operation can significantly increase the pain caused by the downward traction of the tumor. Compared with radial scanning and linear endoscopic ultrasonography, the advantage of microprobe endoscopic ultrasonography is, the microprobe can be introduced using routine gastroscope, it can be used to examine the stenosis region due to its small size, not tending to cause pain and bleeding. All patients successfully completed routine gastroscopy combined with microprobe endoscopic ultrasonography; the internal echo signals of the tumors were effectively evaluated in all cases. However, microprobe EUS images were seriously attenuated and the image quality was low. This is mainly due to the internal tissue of the tumor being mainly fat, so the ultrasonic attenuation is obvious. Microprobe endoscopic ultrasonography often showed good mucosal layer, an irregular submucosal muscle-like area, and slightly hyperechoic areas.

#### 4.2.3. CT

CT and multiplanar reconstruction (MPR) are important methods to evaluate the lesion and have the advantage of convenience and pain-free. CT scan of all cases showed that the density of the tumor was similar to that of fat, and the density of the tumor was heterogeneous and mixed with other ingredients. CT multiplanar reconstruction images can clearly show the size and extent of the lesions. The disadvantage of unenhanced CT is that it cannot distinguish the origin of the lesion. Contrast-enhanced CT was not performed in all six cases, so the role of contrast-enhanced CT in locating the origin of the lesions has not yet been clarified.

### 4.3. Therapeutic Methods

Angiolipomas of the gastrointestinal tract were previously treated by complete resection and could be removed by endoscopy or surgery depending on the location and urgency of the lesion. Surgical excision is preferable to endoscopic excision because of the increased risk of bleeding and perforation for tumor with wider pedicle [13]. Our cases are angiolipoma at the hypopharynx-esophageal introitus; through consulting a large volume of literature, it was found that surgical excision was selected in all the reported cases, local excision under general anesthesia was selected in small pharyngeal lesions [8, 9], and open excision or even esophagectomy was selected in large esophageal lesions [10]; endoscopic minimally invasive treatment was not found in the searched literature. All the cases in our hospital were successfully treated by endoscopic minimally invasive dissection. For pedicled tumors, EMR or ESD resection should be selected for those who meet the conditions of endoscopic surgery, which can reduce surgical risks and postoperative recovery time for patients.

ESD was successfully performed in all 6 patients, which proved that the procedure was safe and reliable. Hemorrhage occurred in all 6 cases, mainly during injection and incision. Hemostasis through forceps-assisted thermocoagulation was successful in all cases; no uncontrollable bleeding occurred. Postoperative wound pain was the main symptom without other postoperative complications. The advantages of ESD include safety, less injury, quick recovery, and no serious complications. The main reason for intraoperative blood loss is that angiolipoma is usually large and rich in blood vessels, especially the vessels at the pedicle. It is easy to damage blood vessels and cause hemorrhage. The best way to prevent and reduce bleeding is more submucosal injection, to increase the distance between blood vessels, to widen the tissue gap, to cut layer by layer, and to use more thermocoagulation forceps to prevent hemostasis. Postoperative pharyngeal pain is considered due to two aspects: First, the wound is located at the pharynx and esophageal introitus, thus often causes traction pain. Second, the wound was stimulated by the digestive juice secreted by the oropharyngeal cavity to produce irritating pain.

Endoscopic mucosal dissection excision for angiolipoma at hypopharynx and esophagus is very difficult. It is mainly due to: First, the blood vessels of the lesions are rich and easy to bleed. Second, it is difficult to control the endoscopic field of vision. Third, the tumor is too big to be taken out. Fourth, endoscopic instruments are difficult to operate. The tumors were not taken out in 2 cases. Instead, they were removed, cut into pieces and moved to the stomach. The two patients whose tumors were not taken out had no postoperative complications such as intestinal obstruction. The remaining tumor parts were excreted through feces.

The largest wound in these patients did not exceed 1/2 of the circumference of the esophageal introitus, which may be related to the absence of postoperative stricture.

It can be seen from our treatment results that large angiolipoma is prone to have large blood vessels. It tends to cause intraoperative bleeding, difficulty in removing the angiolipoma, increased difficulty of operation, and prolonged operation duration. But there was no obvious difference in postoperative recovery. Therefore, early detection, diagnosis, and treatment are recommended to reduce the risk of endoscopic surgery.

## 5. Conclusions

Angiolipoma occurring at the hypopharynx-esophageal introitus is a rare clinical disease, with intermittent chest pain and dysphagia as the main clinical manifestations. Gastroscopy, CT imaging, and microprobe endoscopic ultrasonography are the main methods of preoperative diagnosis. ESD can be an important, minimally invasive, safe, and effective treatment option for angiolipoma at hypopharynx-esophageal introitus.

## Figures and Tables

**Table 1:** Gastroscopic findings.

Cases	Age (years)	Gender	Size (cm)	Obvious pedicle	Color	Prolapsed position	Tumor surface	Displacement of the glottis
1	55	Male	30	Yes	Yellowish-white	Stomach	Hemorrhage and ulceration	Yes
2	27	Female	22	Yes	Yellowish-white	Lower third of esophagus	Congestion	No
3	48	Male	4.5	Yes	Yellowish-white	Esophageal Introitus	Smooth	No
4	85	Male	12	Yes	Yellowish-white	Middle third of esophagus	Congestion	No
5	72	Male	10.5	Yes	Yellowish-white	Middle third of esophagus	Congestion	No
6	29	Female	3.8	Yes	Yellowish-white	Esophageal Introitus	Smooth	No

**Table 2:** Treatment outcomes of 6 patients.

Cases	CT findings	Intraoperative	Tumor taken out	Surgical duration (min)
1	The origin site was visible and the density was heterogeneous	Hemorrhage	Cut into blocks and left in the stomach	14
2	The origin site was not visible and the density was heterogeneous	Hemorrhage	Cut into blocks and left in the stomach	115
3	The origin site was not visible and the density was homogeneous	Hemorrhage	Taken out in whole	58
4	The origin site was not visible and the density was homogeneous	Hemorrhage	Cut into several blocks and taken out	94
5	The origin site was not visible and the density was homogeneous	Hemorrhage	Cut into several blocks and taken out	85
6	The origin site was not visible and the density was homogeneous	Hemorrhage	Taken out in whole	50

## Data Availability

All video data used to support the findings of this study are included within the supplementary information file.

## Supplementary Materials

Supplementary 1 Video 1: submucosal injection of normal saline containing methylene blue and hyaluronic acid at the pedicle of the tumor.

Supplementary 2 Video 2: the mucosa and submucosa layers were gradually dissected with incision knife.

Supplementary 3 Video 3: intraoperative blood vessel bleeding was stopped by means of thermocoagulation forceps, and hemostasis of the dissected wound surface was achieved by thermocoagulation.

Supplementary 4 Video 4: dissection of the tumor.

Supplementary 5 Video 5: the tumor was taken out using a snare.
